# Nemaline Myopathy in a Hypotonic Neonate: Diagnostic Approach for Early Detection and Management

**DOI:** 10.7759/cureus.56866

**Published:** 2024-03-25

**Authors:** Annie Vu, Subah Nanda, Todd Chassee

**Affiliations:** 1 Medicine, Michigan State University College of Human Medicine, East Lansing, USA; 2 Emergency Medicine, Helen DeVos Children's Hospital, Grand Rapids, USA

**Keywords:** multidisciplinary care team, hypoxemia, rapid genetic testing, nemaline myopathy, neonatal hypotonia

## Abstract

Neonatal hypotonia presents with low muscle tone and an array of symptoms that vary depending on the etiology. The differential diagnosis for this condition is complex. It is crucial to exclude life-threatening causes before following a diagnostic algorithm and performing additional tests. Given the wide range of clinical symptoms and etiologies for neonatal hypotonia, rapid genetic testing has the potential to expedite diagnosis, reduce invasive testing such as muscle biopsy, reduce hospital stays, and guide condition management.

A four-week-old girl was admitted to the emergency department (ED) with a one-day history of lethargy, poor feeding, congestion, cough, and hypoxemia. Given positive rhino-enterovirus testing and high inflammatory markers, antibiotics were administered. Imaging, venous blood gas, and blood cultures were negative, and the patient was admitted to the pediatric intensive care unit (PICU) for hypoxemia. After speech-language pathology (SLP) and occupational therapy (OT) evaluation, weak orofacial muscles and feeding issues resulted in a nasogastric tube placement. A swallow study revealed decreased pharyngeal contraction and post-swallow liquid residue. Laryngoscopy showed mild laryngomalacia and dysphagia with aspiration. Genetic testing identified an ACTA1 mutation and confirmed nemaline myopathy (NM). The patient's oxygen levels dropped further during sleep, resulting in diagnoses of severe obstructive and moderate-severe central sleep apnea. Treatment included oxygen therapy, SLP, physical therapy, albuterol, and cough assists. After discharge, the patient was frequently re-admitted with chronic respiratory failure and bronchiolitis and later had gastrostomy and tracheostomy tubes inserted.

This specific case highlights the importance of implementing a diagnostic algorithm for neonatal hypotonia. It is also important for physicians, especially emergency medicine (EM) providers, to first exclude infection, sepsis, and cardiac and respiratory organ failure before looking into other tests. Then, physicians should evaluate for more rare etiologies. In this patient’s case, the hypotonia was due to a rare genetic disease, nemaline myopathy, and a multidisciplinary approach was used for this patient’s care.

## Introduction

Infants with hypotonia manifest with decreased muscle tone and a host of other clinical symptoms that vary based on the disease etiology [1]. The wide array of clinical presentations and potential etiologies complicates both the diagnosis and management of this condition. The differential diagnosis for neonatal hypotonia includes central nervous system and peripheral nervous system etiologies [1-4]. Diagnosing and treating hypotonia in children involves multiple factors (e.g., pattern, family history, presentation) [1]. Physicians should first rule out infectious, metabolic, and encephalopathic causes, including but not limited to sepsis, hypothyroidism, hypoglycemia, and hypoxic-ischemic encephalopathy, to name a few [1]. Metabolic causes may also include peroxisomal disorders and inborn errors of metabolism diseases [1].

Next, a comprehensive history should be taken, including a detailed family history of neuromuscular diseases, developmental delay, and premature death [2]. Details of the pregnancy, including the prenatal, delivery, and postnatal period can also be important [5]. For instance, it could be relevant to ask about prenatal risk factors such as teratogen exposure, maternal diseases like diabetes, abnormal fetal movements, and fetal presentation [5]. A history of congenital infections such as toxoplasmosis, other, rubella, cytomegalovirus, and herpes simplex virus (TORCH) increases the risk for a CNS cause [6-7]. The delivery history, including any complications, along with the appearance, pulse, grimace, activity, and respiration scores (APGAR) should be meticulously recorded in the chart to allow for review [5]. Furthermore, abnormal results from the newborn screen are typically communicated to the pediatrician designated on the screen; however, these results may occasionally go unnoticed, emphasizing the importance of seeking updates if necessary [8].

A developmental assessment and trend of the patient would be useful to assess for motor delay [2]. Inquiring about the dietary and feeding history from caregivers can also yield valuable insights, particularly because neuromuscular junction diseases may manifest as challenges with sucking and swallowing that exacerbate with repetition [2]. For example, specific questions regarding the consumption of honey or canned goods can help diagnose infant botulism [9]. A thorough physical exam should be conducted along with the history taking. The physician needs to establish whether the patient is exhibiting weakness in addition to an abnormal tone or if the patient typically has normal strength but is currently hypotonic [10]. If possible, it is recommended that the physician conduct multiple examinations of the patient to mitigate the influence of potential confounding factors such as variations in patient alertness [10].

This should then be followed by imaging and labs related to the presentation. Neuroimaging can be a valuable tool for determining the cause, as computed tomography (CT) and magnetic resonance imaging (MRI) can identify structural malformations, neuronal migration defects, and brain stem and cerebellar abnormalities [11]. Genetic causes of neonatal hypotonia include chromosomal abnormalities, such as Down syndrome or Prader-Willi syndrome, which can be disclosed via karyotype analysis [1,6]. The use of trio exome and genome sequencing, in which both parents’ genetic sequences are compared with that of the infant can also expedite a diagnosis and is now recommended as a first-line testing option [1]. Other bloodwork including a full blood count, electrolytes, and inflammatory markers can help rule out systemic causes like sepsis and electrolyte imbalances [6]. A creatine kinase test can be useful to rule out muscle, nerves, or neuromuscular junction diseases [6]. A screen for hypothyroidism would be indicated if the etiology of the hypotonia is unclear [2].

## Case presentation

A four-week-old female with two other healthy older siblings, and an unremarkable birth and newborn screening presented to the emergency department (ED) with a one-day history of lethargy, poor feeding, congestion, coughing, and hypoxemia with oxygen saturations at 90%. A chart review of primary care visits revealed documentation of weight loss, hypotonia, weak suck and cry, fatigue, and wheezing during feeds at the two-week well-child visit. At this appointment, the patient was below her birth weight, and the physician discussed with the patient's family the possibility of hospital admission if there was no improvement in weight and advised them to seek emergency care if the patient exhibited signs of lethargy, decreased urine output or other feeding difficulties. The patient returned for a follow-up appointment three days later, during which she showed a weight gain of 14 grams/day, but still exhibited reduced muscle tone. There was a conversation regarding a referral to pediatric neurology for further investigation into central hypotonia at the subsequent follow-up appointment scheduled for a weight check in three days. At this appointment, the patient’s weight had returned to that recorded at the two-week well-child visit, with complaints of respiratory distress and coughing. With a drop in oxygen saturation from 99% to 90%, and worsening muscle tone, there were concerns for respiratory compromise.

As a result, emergency medical services were called to transport the patient to the ED. At the ED, the patient was afebrile at 36.9°C, normotensive at 89/56, with a regular heart rate at 165, regular respiratory rate at 42, and saturating at 96% O2 on high flow nasal cannula at 6L with a fraction of inspired oxygen (FiO2) at 21%, after previous oxygen saturations were in the 70s. The patient tested positive for rhino-enterovirus with leukocytosis, elevated C-reactive protein (CRP), and elevated procalcitonin (Table 1). As per sepsis protocol, ampicillin and gentamicin were administered. Chest X-ray (CXR), electrocardiogram, head CT without contrast, venous blood gas, and blood cultures were unremarkable (Table 1). 

**Table 1:** Initial laboratory values pCO2 – partial pressure of carbon dioxide, pO2 - partial pressure of oxygen

Lab	Value	Reference range
White blood count (WBC)	24.85 x10*3/uL	6.00-20.00 x10*3/uL
C-reactive protein (CRP)	15.5 mg/L	< = 5.0 mg/L
Procalcitonin	0.28 ng/mL	< = 0.25 ng/mL
Rhinovirus-enterovirus polymerase chain reaction (PCR) film array	Detected	Not Detected
Venous blood gas		
pH	7.37	7.32-7.42
pCO2	52 mm/Hg	38-52 mm/Hg
pO2	31 mm/Hg	24-48 mm/Hg
Oxygen saturation	56%	65-100%
Bicarbonate	30 mmol/L	20-28 mmol/L
Base excess	3.0 mmol/L	2.0-2.0 mmol/L
Venous blood culture	Negative	Negative
CSF culture	Negative	Negative
Glucose CSF	43 mg/dL	45-80 mg/dL
Protein CSF	306 mg/dL	20-70 mg/dL
Thyroid-stimulating hormone (TSH)	1.17 mcIU/mL	0.80-8.00 mcIU/mL
Creatine kinase (CK)	63 U/L	20-175 U/L
Organic acids screen, random urine	No unusual organic acids	No unusual organic acids
Ammonia	43 umol/L	0-60 umol/L
Homocysteine, total, plasma level	5 mcmol/L	0-13 mcmol/L
Lactic acid	0.9 mmol/L	0.0-2.0 mmol/L
Pyruvic acid	0.08 mmol/L	0.03-0.15 mmol/L
Acylcarnitine	11 nmol/mL	4-15 nmol/mL
Total carnitine	60 nmol/mL	19-59 nmol/mL
Free carnitine	49 nmol/mL	12-46 nmol/mL

The patient was admitted to the pediatric intensive care unit (PICU). Speech-language pathology and occupational therapy evaluation revealed weakened orofacial muscles, intolerance of secretions, and aspiration with feeds, so a nasogastric tube was placed. A full CSF workup could not be performed, as interventional radiology (IR) could only extract 2 mL of bloody CSF. The limited CSF results showed high protein and low glucose, with no bacterial growth on culture (Table 1). As CNS infection was ruled out, further genetic and metabolic workup was warranted according to the pediatric neurologist. Family history was remarkable for a history of thyroid issues, but the patient’s mom received prenatal care and the patient’s TSH was normal at birth. Furthermore, thyroid-stimulating hormone (TSH), creatine kinase (CK), urine organic acid, ammonia, homocysteine, lactic acid, pyruvic acid, acylcarnitine, free and total carnitine levels were unremarkable (Table 1). Whole genome sequencing was sent one day after the patient’s ED visit and six days later, revealed an ACTA1 mutation, revealing a diagnosis of nemaline myopathy (NM) (Table 2).

**Table 2:** Whole genome sequencing results revealing the ACTA1 mutation, consistent with nemaline myopathy

Gene	Variant	Condition	Zygosity (inheritance)	Variant classification
ACTA1	c.808G>A p.Gly270Ser	ACTA1-related disorders	Heterozygous (de novo)	Pathogenic

Two days later, the patient was taken off oxygen and moved from the PICU to the general pediatric floor. Vomiting occurred during feeding trials, and a subsequent swallow study showed reduced pharyngeal contraction and mild-moderate post-swallow residue of thick liquids. Blood culture and CSF remained negative, and antibiotics were discontinued. The patient became hypoxic again with saturations dropping to 80-85% while asleep, so CXR and inhaled albuterol were ordered. CXR showed no notable pathology, and the albuterol improved the patient’s latch, seal, and intake of thick liquids (up to 15 mL). However, she remained fatigued. Pulmonology assessed the patient and ordered a polysomnography in the hospital which showed severe obstructive sleep apnea and moderate-severe central sleep apnea. She was then placed on a nasal cannula at 0.5 L/min, chest physical therapy, and cough assists. An echocardiogram ruled out pulmonary hypertension, and an MRI ruled out Chiari malformation as an etiology for her hypoxemia. Laryngoscopy revealed mild laryngomalacia and dysphagia with aspiration, but supraglottoplasty was not recommended. 

The patient was discharged on oxygen after two weeks of hospitalization, after the parents were trained on nasogastric (NG) tube placement and feeding, and educated regarding chest percussion therapy and the new diagnosis of nemaline myopathy. However, the patient continued to require frequent visits to the ED and PICU for chronic respiratory failure and bronchiolitis. Several months later, the patient underwent bronchoscopy, microlaryngoscopy, and a gastrostomy placement, and had a tracheostomy tube inserted. She is currently on heat moisture exchange, speaking valve trials during the day, and night-time ventilator support. 

## Discussion

Hypotonia in children may be caused by infection, genetic syndromes, metabolic disorders, endocrine issues, brain malformations, drug toxicity, neuromuscular disorders, and congenital infections [12] (Table 3).

**Table 3:** Central and peripheral etiologies of neonatal hypotonia

Central hypotonia	
Cerebral	Cerebral palsy, Spinal cord injuries, Cerebral dysgenesis, Intracranial hemorrhage, Hypoxic-ischemic encephalopathy, Congenital cytogenetic abnormalities, Chiari malformation
Genetic	Prader-Willi syndrome, Angelman syndrome, Nemaline myopathy, Congenital hypothyroidism, Hypoglycemia, Congenital adrenal hyperplasia
Chromosomal	Down syndrome, Edwards syndrome (Trisomy 18), Patau syndrome (Trisomy 13)
Inborn errors of metabolism	Tay-Sachs, Congenital disorders of glycosylation, Carnitine cycle defects, Fatty acid oxidation defects, Disorders of creatine metabolism, Glycogen storage diseases, Organic acidemias, Peroxisomal disorders, Urea cycle defects
Infections	Bacterial sepsis (urinary tract infection/pyelonephritis, bacteremia, meningitis, pneumonia, cellulitis, gastroenteritis, septic arthritis, osteomyelitis), Botulism, Viral illness (Herpes simplex virus, Enterovirus, Cytomegalovirus, Bronchiolitis, Influenza)
Toxins	Environmental (carbon monoxide, methemoglobinemia), Drug exposure (heroin, cocaine, phencyclidine, marijuana)
Peripheral and muscular hypotonia	
Anterior horn cell disorders (motor neurons)	Acute infantile spinal muscular atrophy, Traumatic myelopathy, Hypoxic-ischemic myelopathy, Arthrogryposis multiplex congenita
Congenital motor or sensory neuropathies	Charcot-Marie-Tooth disease, Familial dysautonomia, Infantile neuroaxonal degeneration, Congenital hypomyelination neuropathy, Dejerine-Sottas disease, Hereditary sensory and autonomic neuropathy
Neuromuscular junction disorders	Transient-acquired neonatal myasthenia, Congenital myasthenia, Magnesium toxicity, Aminoglycoside toxicity
Skeletal muscle	Duchenne and Becker muscular dystrophy, Classic form of congenital muscular dystrophy, Congenital form of congenital muscular dystrophy (Walker-Warburg disease, Muscle-eye-brain disease, Fukuyama disease), Early infantile facioscapulohumeral dystrophy, Congenital myotonic dystrophy

**Figure 1:**
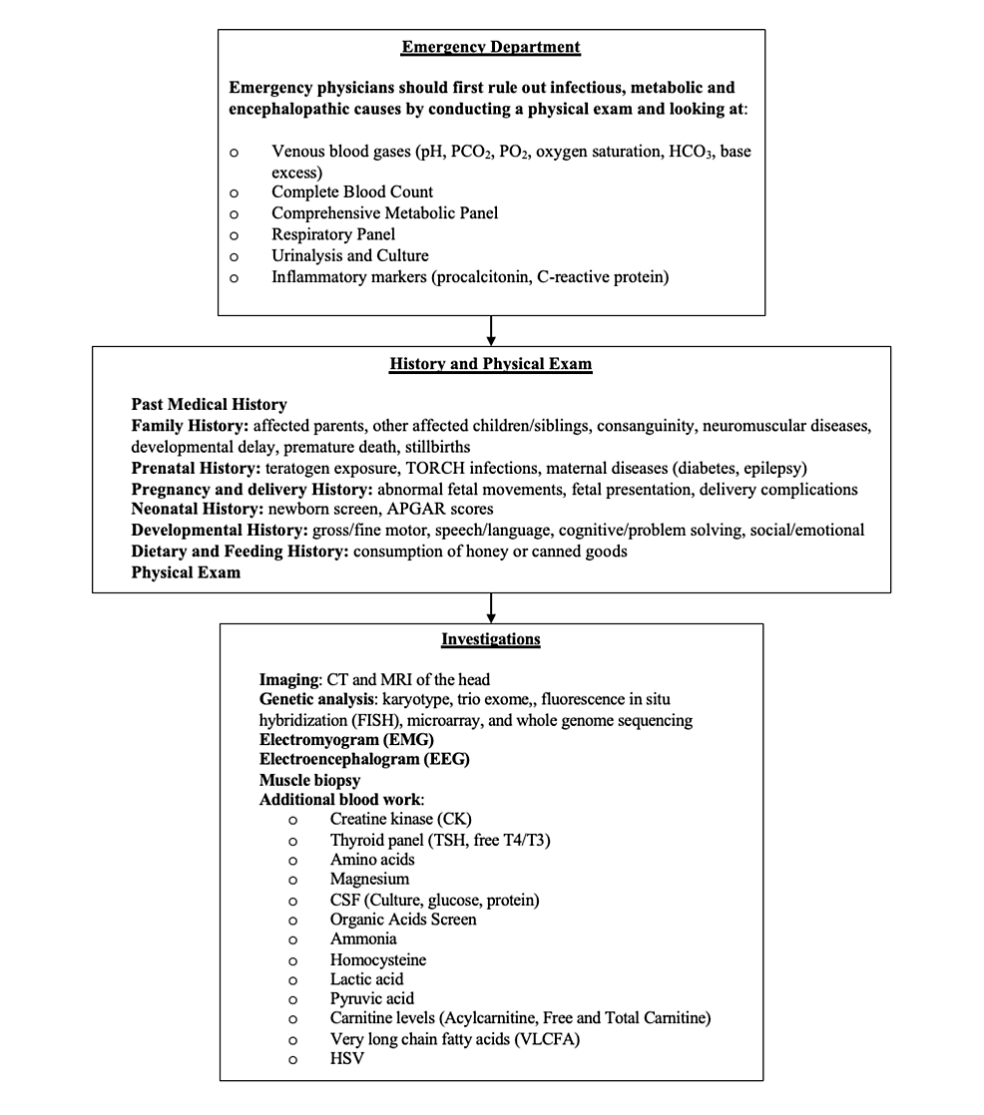
Diagnostic algorithm for a patient presenting with hypotonia pCO2 – partial pressure of carbon dioxide, pO2 - partial pressure of oxygen, HCO3 - bicarbonate, TORCH - toxoplasmosis, other, rubella, cytomegalovirus, and herpes simplex virus, APGAR - appearance, pulse, grimace, activity, and respiration scores

In the ED, secondary causes should be excluded first (e.g. infection, hypoglycemia, metabolic errors, and hypoxic-ischemic encephalopathy) [1] (Figure 1). A thorough family, sibling, pregnancy, and birth history should then be taken [13] (Figure 1). Imaging and labs related to the clinical presentation must be done, along with rapid-trio genome or exome sequencing [1] (Figure 1). If a diagnosis is made, goals of care should be discussed. If no diagnosis is found, karyotype, fluorescence in situ hybridization (FISH), microarray, ammonia, lactate, amino acids, and very long chain fatty acid (VLCFA) tests can be completed [11,13] (Figure 1). Muscle biopsy, nerve conduction studies, electromyography, and mitochondrial genome analysis may also be included [1,14] (Figure 1).

Sepsis was ruled out as the cause of hypotonia, so the patient underwent tests for metabolic, neuromuscular, and genetic disorders. Ultimately, a genetic disorder, nemaline myopathy explained her low muscle tone, feeding problems, and difficulty breathing exacerbated by the respiratory illness. NM is a congenital myopathy that causes generalized muscle weakness, hypotonia, and respiratory issues in 1 out of every 50,000 births [15]. Mutations in 12 genes that encode proteins related to the thin filament of the sarcomere have been identified, with NEB and ACTA1 being the most common [15]. Traditionally, nemaline myopathy is diagnosed by the presence of “nemaline bodies” or “rods” on skeletal muscle biopsy, which appear as small red inclusions arranged in clusters on Gomori trichrome stain [16]. The role of muscle biopsy in the diagnostic workup of hypotonia is controversial as it is invasive, and the results are not specific and may yield inconclusive results [16]. However, advances in molecular genetic testing offer a non-invasive and more accurate alternative to muscle biopsy, which can be painful and carry risks of complications such as bleeding or infection [1,17]. Additionally, genetic testing can be performed earlier in the diagnostic process, allowing for timely intervention and initiation of targeted treatments [1]. This is particularly beneficial in cases where early intervention can significantly improve short-term outcomes and prevent the progression of the disease, ultimately leading to a better long-term prognosis.

The clinical picture of NM is variable, with severity differing between patients with the same mutations in the same gene [15]. The clinical variability observed in NM, despite patients sharing identical mutations with the same gene, underscores the complex interplay of genetic, environmental, and epigenetic factors in shaping disease manifestations [18]. For instance, the onset of severe weakness in utero can lead to intra-uterine growth restriction (IUGR), decreased fetal movement, facial anomalies, and joint contractures [15, 19]. On the other hand, patients with the same mutations in the same gene may present at birth with generalized muscle weakness alone without contractures [15]. Milder forms of NM may present later in childhood with delayed gross motor milestones or signs of muscle weakness [15]. NM can also present in adulthood, as patients may have muscle weakness, respiratory failure, myopathic facies, high-arched palates, small tongues and mouths, and thin ribs [15]. The diversity in disease presentation can pose a significant challenge in diagnosing NM and tailoring effective treatment strategies. As there is no cure for NM, treatment focuses on managing symptoms, preserving muscle strength, and aiding respiratory function [15].

This case report demonstrates the importance of early detection and diagnosis in the context of genetic disorders, especially if there are specific treatments available. Identifying genetic conditions, like nemaline myopathy, early in life can lead to proactive interventions and therapies to minimize disease severity and progression. Although there is currently no cure for nemaline myopathy, the patient’s early diagnosis and access to a multi-disciplinary care team composed of respiratory, occupational, and physical therapists will help improve her disease course and progression. This collaborative effort ensures that the patient receives comprehensive supportive care aimed at improving her quality of life.

## Conclusions

In conclusion, this case underscores the complexity of diagnosing and managing neonatal hypotonia, a condition with a myriad of potential primary and secondary etiologies. Early diagnosis, as exemplified by the identification of nemaline myopathy (NM) in this case, is important as it facilitates the timely initiation of tailored interventions aimed at symptom management and disease progression. While NM poses significant challenges due to its lack of cure, the proactive involvement of a multidisciplinary care team offers the patient opportunities for optimized care, potentially improving her quality of life and long-term outcomes. A multidisciplinary team of pediatricians, medical geneticists, neurologists, pulmonologists, and occupational, respiratory, and physical therapists is essential for assessing and managing hypotonia.
